# Overcoming the biological aging of titanium using a wet storage method after ultraviolet treatment

**DOI:** 10.1038/s41598-017-04192-9

**Published:** 2017-06-19

**Authors:** Sung-Hwan Choi, Won-Seok Jeong, Jung-Yul Cha, Jae-Hoon Lee, Kee-Joon Lee, Hyung-Seog Yu, Eun-Ha Choi, Kwang-Mahn Kim, Chung-Ju Hwang

**Affiliations:** 1 Department of Orthodontics, Institute of Craniofacial Deformity, College of Dentistry, Yonsei University, Seoul, 03722 Korea; 2 Department and Research Institute of Dental Biomaterials and Bioengineering, BK21 PLUS Project, College of Dentistry, Yonsei University, Seoul, 03722 Korea; 3 Department of Prosthodontics, College of Dentistry, Yonsei University, Seoul, 03722 Korea; 4 Plasma Bioscience Research Center, Kwangwoon University, Seoul, 01897 Korea

## Abstract

We evaluated whether the biological activity of the surface of titanium, when stored in an aqueous solution after ultraviolet (UV) treatment, is comparable to that of the surface immediately after UV treatment. We subjected Grade IV titanium discs with machined surfaces to UV radiation for 15 min and then tested them immediately and after storage for 28 days, with and without distilled H_2_O (dH_2_O). We evaluated the surface characteristics using surface profiling, contact angle analysis, X-ray photoelectron spectroscopy, and in terms of the surface zeta-potential. We determined the level of biological activity by analysing albumin adsorption, MC3T3-E1 and human mesenchymal cell adhesion and cytoskeleton development, as well as the production of intracellular reactive oxygen species between groups. The surface characteristics produced by the UV irradiation were maintained in dH_2_O for 28 days. We found that titanium stored in dH_2_O for 28 days after UV treatment exhibited enhanced protein adsorption, cell attachment, and cytoskeleton development. Titanium stored in dH_2_O for 28 days after UV irradiation exhibited a lower level of oxidative stress, comparable to that of the titanium immediately after UV treatment. UV treatment combined with wet storage can be used as a means of overcoming the biological aging of titanium.

## Introduction

Titanium is widely used commercially as an orthopaedic and dental implant material for the rehabilitation of degenerated joint function and the restoration of missing teeth. It is used because titanium dioxide exhibits high biocompatibility and excellent corrosion resistance, and can also achieve osseointegration, defined as a structurally and functionally direct connection between the surrounding bone and implant surfaces without fibrous tissue formation^1, 2^. To date, to increase the clinical success rate for high-risk patients with bone that has been compromised by aging or diseases such as diabetes, various surface modifications have been developed, combining micro- and nanoscale features to mimic the characteristics of natural bone^3–5^.

However, previous studies have reported that the time-dependent degradation of the osteoconductivity of a titanium surface, regardless of the surface topography, led to a decrease in the biomechanical strength of the bone–titanium integration, relative to a newly prepared titanium surface, because most commercially used titanium implants are provided in sterile, gas-permeable packaging, such that they can be stored for extended periods (typically up to 5 years)^6^. Att *et al*.^7^ reported that the biological aging of a titanium surface was associated with a greatly reduced ability to attain protein and osteogenic cell adhesion due to the progressive accumulation of hydrocarbons on the titanium surface under ambient conditions. As the percentage of hydrocarbons on a titanium surface increases, the surface zeta potential on the surface changes from electropositive to electronegative, while a cell-attracting terminal consisting of an Arg-Gly-Asp (RGD) sequence can be blocked by the hydrocarbons^8–10^. Thus, this phenomenon prevents negatively charged blood protein and the extracellular matrix of the cells from attaching to the implant surface.

Recently, ultraviolet light (UV) has been shown to be capable of changing the physicochemical properties of a titanium surface from hydrophobic to hydrophilic, due to the removal of the surface hydrocarbons and/or the formation of reactive hydroxyl radical species with a reduced surface negative charge without altering the surface topography^11, 12^. Aita *et al*.^13^ reported that, in an animal model, the UV treatment of titanium surfaces markedly increased their osteoconductivity while also enhancing new bone formation, the bone–implant contact, and the strength of the bone–titanium integration, relative to untreated titanium.

However, most previous studies have only investigated the biological effects of titanium implants immediately after their UV treatment, assuming their chairside use, because the efficacy of this treatment has long been known to be lacking^14, 15^. Miyauchi *et al*.^16^ reported that the effects of UV treatment could be maintained for only a few minutes after the irradiation in ambient air at room temperature. Choi *et al*.^9^ reported that, when the titanium was stored in a sealed container at room temperature for 4 weeks after UV treatment, the residual biological effects could not promote osteoblast cell attachment and cytoskeleton development over time, relative to untreated titanium. In a clinical situation, it would not be practical for a physician to apply UV treatment to a titanium implant for 15 min to 48 h immediately before the surgery, and its sterility may be compromised at the instant it is removed from its package prior to the UV treatment being done chairside^13, 15, 17, 18^. However, if appropriate storage strategies are applied after the UV treatment of titanium implants, the hydrophilicity of the titanium surface could be maintained at a level similar to that observed immediately after the treatment regardless of the duration of the storage, and the commercially available pretreatment of titanium implants with UV may be applicable. Recently, the storing of titanium implants in isotonic saline or distilled water immediately after their manufacture has been shown to prevent fouling by organic impurities while maintaining their hydrophilicity^6, 19, 20^. Shen *et al*.^21, 22^ reported that UV treatment can further enhance the already increased bioactivity of the surface of a titanium implant stored in distilled water and the effect of UV treatment may be much more overwhelming than that of the storage medium. However, the study did not assess the consistency of the validity of the combined effects over time because it assumed that the application of a UV treatment is possible only immediately before implant placement, in the same way as the studies mentioned above^13, 15, 17, 18^. Kim *et al*.^23^ reported that UV combined with alendronate soaking could synergistically enhance the biological activity of titanium implant surfaces, both *in vitro* and *in vivo*. However, the study did not suggest a period of soaking titanium in alendronate solution without presenting standardised storage protocols. To the best our knowledge, few studies have evaluated whether the time-dependent biological activity of the surface of titanium, stored in an aqueous solution after UV treatment, is comparable to that of the surface immediately after UV treatment for commercially available UV-pretreated titanium implants.

Therefore, we chose to focus on UV treatment and wet-storage as solutions for preventing contamination by hydrocarbons while maintaining the surface hydrophilicity of the titanium implants during storage. In this study, we set out to evaluate whether the biological activity of the titanium surface, when stored in an aqueous solution after UV treatment, is comparable to that immediately after UV treatment.

## Materials and Methods

### Preparation of titanium samples

We prepared disc-shaped titanium samples (12.0 mm diameter, 1.0 mm thickness) by machining commercially available pure titanium (grade IV; Osstem Implant Co., Seoul, Korea). We cleaned the titanium discs with acetone, then alcohol, and then distilled water for 15 min each, using an ultrasonic cleaner. We then sterilised them using ethylene oxide (EO) gas at a temperature of 55 °C for 1 h^24, 25^. The prepared titanium discs were fully aged by storing in sealed 12-well cell culture plates under dark ambient conditions at room temperature for at least 8 weeks^26, 27^. After the end of the aging period, we divided the titanium samples into three groups for further processing:

- Immediately: The titanium specimens were irradiated by UV under ambient conditions. The UV irradiation was applied for 15 min using a commercially available photo device (TheraBeam Affiny; Ushio Inc., Tokyo, Japan). The UV was delivered as a mixed spectrum using a UV lamp. The measured intensities were 0.05 mW/cm^2^ (λ = 360 ± 20 nm) and 2 mW/cm^2^ (λ = 250 ± 20 nm)^9, 28^.
- UV-dry: Immediately after the UV treatment, the specimens were stored in sealed 12-well cell culture plates under sterile humidified conditions at 37 °C in 5% CO_2_ for 28 days to allow sufficient aging before the specimen was subjected to testing^10^.
- UV-wet: Immediately after UV treatment, the specimens were stored in sealed 12-well cell culture plates with dH_2_O under sterile humidified conditions at 37 °C in 5% CO_2_ for 28 days. The dH_2_O was chosen as the wet storage medium in this study to avoid the influence of heterogeneous ions. The samples stored in dH_2_O were blown dry in a stream of nitrogen prior to the experiments^20, 21^.

### Surface characterization

To investigate the change in the surface roughness of the titanium sample before and after UV irradiation, we examined the surface morphologies of the samples using an optical three-dimensional surface profiler (ContourGT; Bruker, AZ, USA) using the vertical scanning interferometry (VSI) mode with a green luminous source. We also measured the average surface roughness at a magnification of 10× with a scanning area of 310 µm × 230 µm. The titanium surfaces were examined using a UV-Vis-NIR spectrophotometer (Cary5000, Agilent Technologies, CA, USA) to determine their optical properties.

To investigate the difference in the hydrophilicity of the titanium disc surface between the groups, immediately, 7, and 28 days after UV treatment, with and without dH_2_O, we assessed the wettability by measuring the contact angle of a 4-µL dH_2_O droplet placed on the centre of each sample surface. Then, 10 s after we had placed the drop on the surface, we measured the contact angle using a video contact angle goniometer (Phoenix 300; SEO, Gyeonggi-do, Korea) with Image XP software (SEO). We evaluated the chemical composition of the titanium disc surface of the groups, immediately, 7, and 28 days after the UV treatment, using X-ray photoelectron spectroscopy (XPS; K-alpha; Thermo VG, UK) and a monochromatic Al Kα line (1486.6 eV) with the following parameters: 12 kV, 3 mA, and a spot size of 400 µm. We examined the titanium, oxygen, and carbon contents in a vacuum at each time point.

### Zeta potential

To investigate the difference in the zeta potential of the titanium disc surface between the groups, immediately and 28 days after UV treatment, as well as with and without dH_2_O, we dispersed the samples together with monitoring particles (polystyrene latex) in a high-purity silica glass cell. This glass cell was connected to a laser electrophoresis spectroscope (ELSZ 1000; Otsuka Electronics Co., Osaka, Japan) to measure the zeta potential of the surface^9, 29^. The electrophoretic mobility or zeta potential is highly dependent on both the sample surface and the medium in which it is immersed. By measuring the zeta potential of the monitoring particles as a function of the distance from the surface under investigation, we could obtain the surface zeta potential. We performed these measurements in a 10-mM NaCl solution at pH 7.4. We selected the data according to when the distribution of the zeta potential according to the height of the cuvette was parabolic relative to the centre. The electrokinetic streaming potential was automatically calculated using the Smoluchowski method.

### Protein adsorption assay

We used bovine serum albumin, fraction V (BSA; Pierce Biotechnology, Inc., IL, USA) as a model protein. We pipetted and spread the protein solution (100 µL; 1 mg/mL in phosphate-buffered saline [PBS], pH 7.4) onto each sample surface, immediately and 28 days after UV treatment, with and without dH_2_O. After 4 h of incubation under sterile humidified conditions at 37 °C in 5% CO_2_, we removed any nonadherent protein by washing twice with PBS. We measured the amount of protein by using 200 µL of microbicinchoninic acid (Pierce Biotechnology, Inc., IL, USA) followed by incubation at 37 °C for 30 min^7, 13^. The optical density (OD) of each sample was then quantified using a microplate reader (Epoch, BioTek Instruments, VT, USA) at 562 nm.

### Cell culture

We used Murine MC3T3-E1 osteoblast cells (CRL-2593; American Type Culture Collection, VA, USA) at passage 7 to determine the cellular responses to the treatments. The cells were cultured in an alpha-MEM cell culture medium (Gibco, NY, USA) containing 10% foetal bovine serum (FBS; Gibco), penicillin (100 U/mL; Gibco), and streptomycin (100 mg/mL; Gibco) at 37 °C in 5% CO_2_. After the cells had reached 80% confluence, we detached them using 0.25% trypsin/1-mM EDTA-4Na (Gibco) to prevent contact inhibition.

We acquired human mesenchymal stem cells (hMSC) (Lonza, Allendale, NJ, USA). We maintained the passages of the cells between 2 and 4. As the culture medium, we used Dulbecco’s modified eagle medium (DMEM; Gibco), supplemented with 10% human foetal bovine serum (FBS; Gibco), and 1% antibiotic-antimycotic (Gibco) at 37 °C in 5% CO_2_. We changed the cell culture medium every 48 h.

### Cell adhesion assay

We placed a total of 1 × 10^4^ MC3T3-E1 and hMSC cells in 100 µL onto the surfaces of each of the samples in a 12-well plate, immediately and 28 days after UV treatment, with and without dH_2_O. After these samples had been incubated for 4 h, these quantifications were performed using water-soluble tetrazolium salt (WST)-based colorimetry (EZ-1000; DoGenBio Co., Gyeonggi-do, Korea). We then incubated the cells at 37 °C for 1 h with tetrazolium salt (WST) reagent, and measured the amount of formazan product using a microplate reader (Epoch; BioTek Instruments) at 450 nm.

### Cell morphology and morphometry

We placed a total of 1 × 10^4^ MC3T3-E1 cells in 100 µL on the surface of each sample in a 12-well plate, immediately and 28 days after the UV treatment, with and without dH_2_O. After incubating the cells on the treated titanium disc surfaces for 4 h at 37 °C in 5% CO_2_, we stained the cells using diamidino-2-phenylindole, dihydrochloride (DAPI; blue for the nuclei; Molecular Probes, Invitrogen, NY, USA) and rhodamine phalloidin (red for the F-actin filaments; Molecular Probes). We then applied confocal laser-scanning microscopy (LSM 700; Carl Zeiss, Jena, Germany) to examine the cell morphology and cytoskeletal arrangement. We randomly selected twelve single cells with typical morphology features from three different points on the titanium surface^30^. We then performed a quantitative assessment of the cell area using the ImageJ software (NIH, Bethesda, MD, USA).

### Intracellular ROS level

To investigate the oxidative stress generated while the cells attach to the titanium surface, we placed a total of 1 × 10^4^ hMSC cells in 100 µL on the surface of each sample in a 12-well plate, immediately and 28 days after UV treatment, with and without dH_2_O. After we had incubated the cells on the treated titanium disc surfaces for 24 h at 37 °C in 5% CO_2_, we detected the generation of intracellular ROS levels using 2′, 7′-dichlorofluorescin diacetate (DCF-DA; Invitrogen, Carlsbad, CA) fluorophotometry. We incubated the cells with 100-µM DCF-DA for 1 h at 37 °C in 5% CO_2_. The peak excitation wavelength for the oxidised DCF was 492 nm, while for the emission, it was 527 nm. We used confocal laser-scanning microscopy to observe the intracellular ROS signal, while the fluorescence density was quantitatively determined using the ImageJ software (NIH, Bethesda, MD, USA).

### Statistical analysis

For all the statistical analyses, we used IBM SPSS software, version 21.0 (IBM Korea Inc., Seoul, Korea) for Windows. Based on the results of previous studies^8, 24, 31^, we used at least four samples in each experiment, repeating each experiment three times. We analysed the results obtained for the three groups at each time point by means of one-way analysis of variance (ANOVA) using Tukey’s method. Differences in the *P* values of less than 0.05 were considered statistically significant.

## Results

### Surface characterization

**Figure 1:**
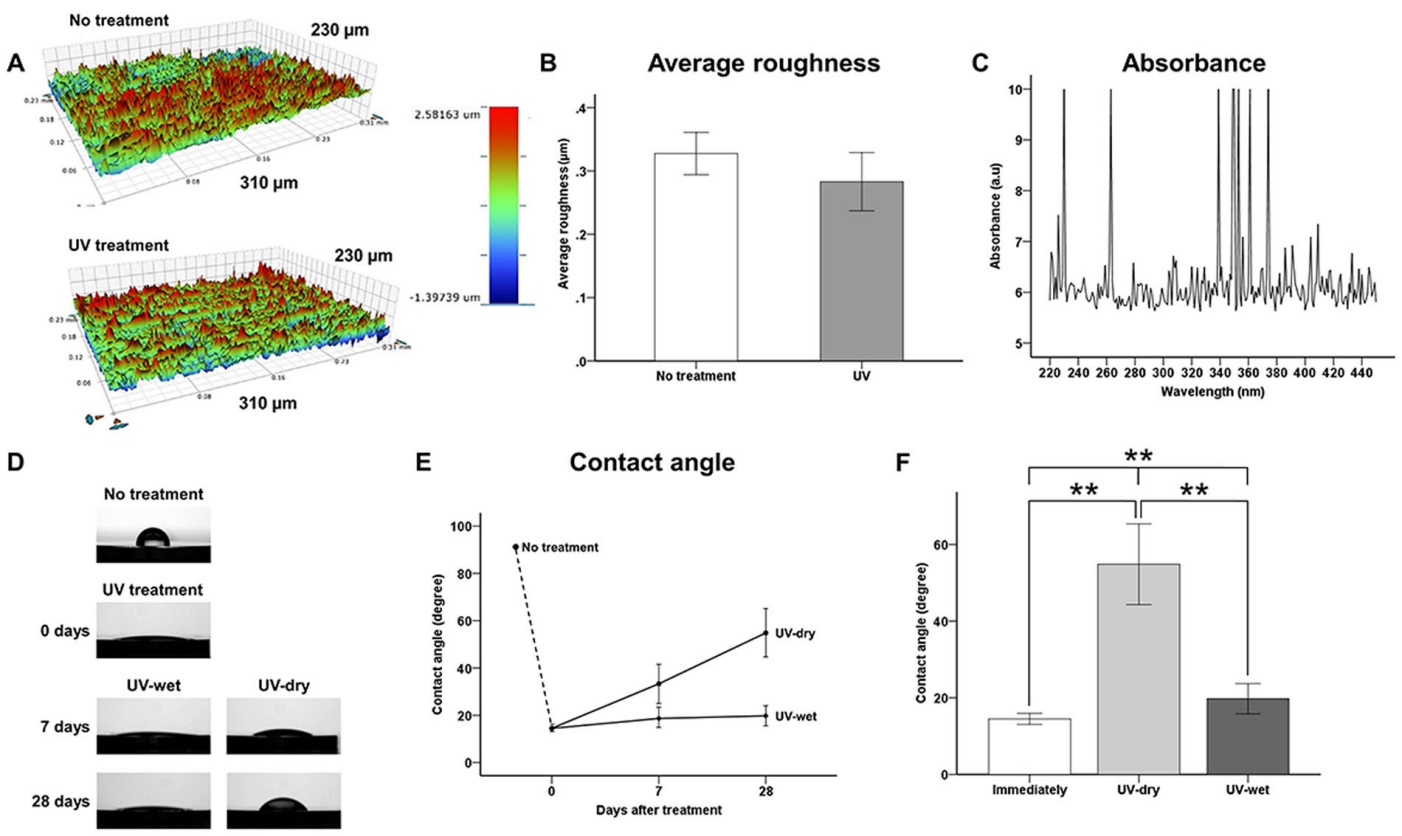
Surface characteristics of titanium discs. (**A**) Three-dimensional surface topographic images of surfaces of untreated titanium discs and immediately after UV treatment. (**B**) Surface average roughness quantitatively measured at a magnification of 10× with a scanning area of 310 µm × 230 µm. (**C**) Optical properties (absorbance units) of titanium surfaces as obtained with UV-Vis-NIR spectrophotometer. (**D**) Comparison of wettability by water, as measured using a 4-µL d H_2_O droplet at the centre of each sample surface, for each group. (**E**,**F**) Comparison of contact angle with the titanium disc surface, for each group. UV-dry, immediately after UV treatment, specimen stored in sealed 12-well cell culture plates under sterile humidified conditions at 37 °C in 5% CO_2_ for 28 days. UV-wet, immediately after UV treatment, specimen stored in sealed 12-well cell culture plates with dH_2_O under sterile humidified conditions at 37 °C in 5% CO_2_ for 28 days. **P < 0.01 for comparisons between groups.

The UV-treated titanium discs showed no marked differences in their average surface roughness relative to the untreated titanium discs under three-dimensional surface analysis (Fig. 1A,B). The average roughnesses of the untreated and UV-treated titanium discs were 0.33 ± 0.03 µm and 0.28 ± 0.05 µm, respectively (*P* > 0.05). UV–vis absorption spectra for titanium surfaces showed the absorption peaks mainly appeared at around 360 nm (339–374 nm). It was considered that titanium surface was mainly reacted in the UV-A wavelength ranges (315–380 nm) (Fig. 1C).

There was a significant difference in the wettability by water between the groups (*P* < 0.01; Fig. 1D–F). As shown in Fig. 1D, the dH_2_O droplet did not spread and maintained its arc shape on the surface of the untreated titanium disc. However, the water droplets on the titanium surfaces immediately after UV treatment, and on the UV-wet surfaces 28 days after treatment, had spread so much that they were difficult to identify. Immediately after UV treatment, the contact angle of the titanium surface shifted from 89.56 ± 3.97° to 14.49 ± 1.44°. At 7 days and 28 days after the UV treatment, the contact angles on the UV-dry titanium discs had shifted from 33.30 ± 8.16° to 54.83 ± 10.54°, respectively. These values were significantly higher than those on the titanium surface immediately after UV treatment and on the UV-wet titanium surface at each time point (*P* = 0.001). The contact angles on the UV-wet titanium discs had shifted from 18.65 ± 4.23° to 19.76 ± 3.94°, at 7 days and 28 days after UV treatment, respectively. There were no significant differences in the contact angles on the titanium surface immediately after UV treatment and on the UV-wet titanium surfaces, regardless of the storage time (*P* > 0.05).

### Changes in surface chemical compositions immediately, 7 days, and 28 days after UV treatment with and without dH_2_O

**Figure 2:**
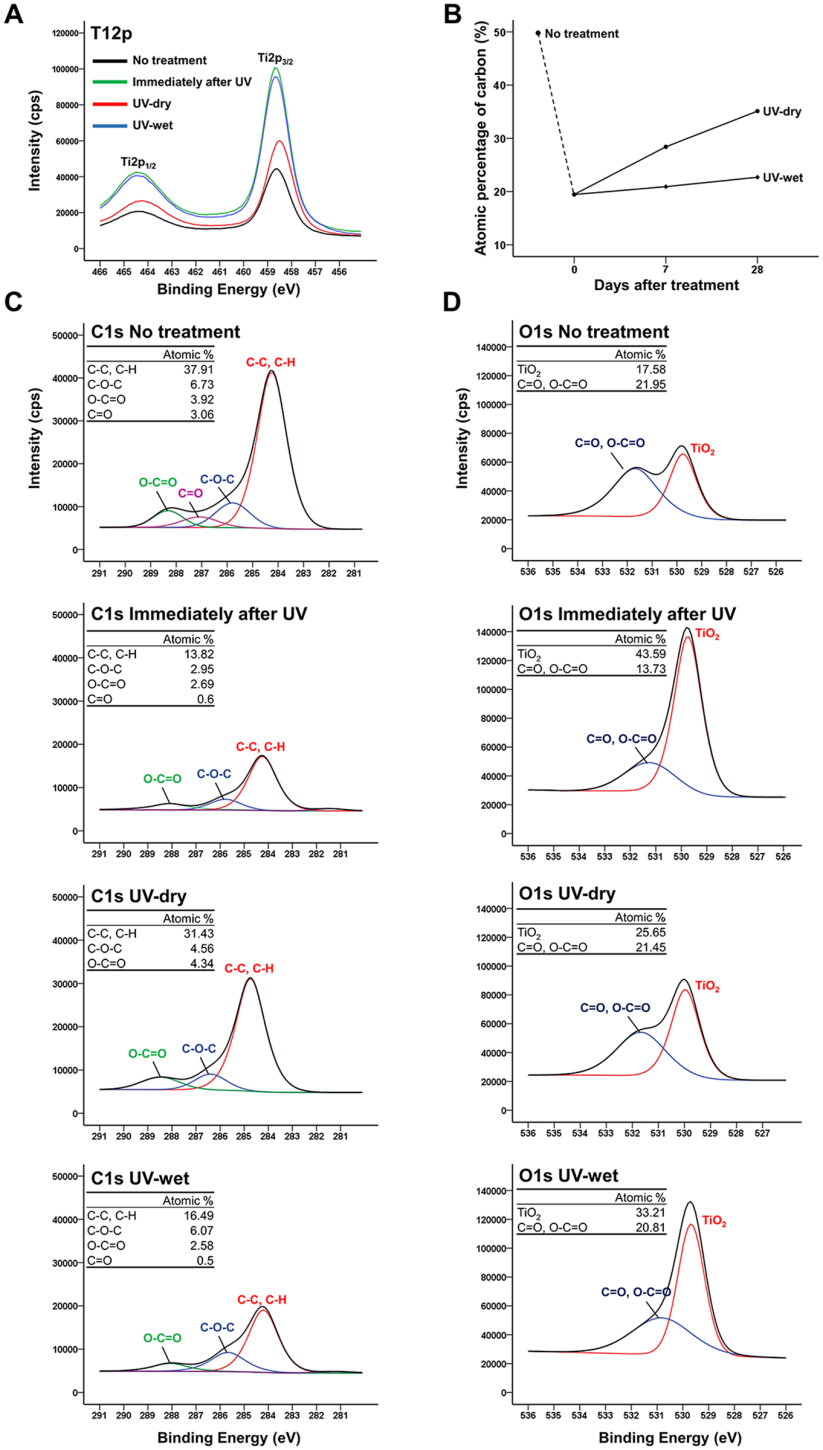
(**A**) Ti2p spectra immediately and 28 days after treatment, for each group. (**B**) Changes in atomic percentages of carbon over time after treatment, for each group. Changes in spectra and atomic percentages of C1s (**C**) and O1s (**D**) immediately and 28 days after treatment, for each group.

As shown in Fig. 2A, the major peaks corresponding to the Ti 2p 1/2 and Ti 2p 3/2 components were located at binding energies of 458.7 to 464.3 eV. The normalised atomic concentration of titanium was found to be in the following order: Immediately after UV treatment (22.63%) >UV-wet (20.17%) >UV-dry (12.06%) >untreated titanium surface (8.85%). The carbon content immediately after UV treatment had changed considerably, from 49.48% to 19.45% (Fig. 2B). The carbon content on the UV-wet titanium surface increased slightly from 20.92% to 22.69% at 7 days and 28 days after the UV treatment, respectively, but those of the UV-dry titanium surface increased from 28.41% to 35.12% at 7 days and 28 days after the UV treatment, respectively. The carbon-related peaks (C-C, C-H at a binding energy of 284.8 eV, C-O-C at a binding energy of 286.3 eV, C=O at a binding energy of 287.8 eV, and O-C=O at a binding energy of 288.8 eV) were lower than that for the UV-treated titanium surface, compared with those of the untreated titanium surfaces, regardless of whether they had been stored in dH_2_O (Fig. 2C). All the C1s peaks, regardless of their group, had a symmetrical shape and high concentrations of sp^3^-bonded carbon. The peak intensities corresponding to the carbon of the UV-wet surface remained similar to those of the titanium surface immediately after UV treatment. The peaks corresponding to the TiO_2_ at a binding energy of 530.1 eV on the titanium surface immediately after UV treatment and on the UV-wet titanium surface were higher than those for the UV-dry and untreated titanium surfaces. Similar to the carbon-related peaks, UV treatment removes carbon pollutants on the titanium surface and the peaks corresponding to C=O, C-C=O at a binding energy of 532.2 eV on the titanium surface immediately after UV treatment, as well as on the UV-wet titanium surface, were lower than those for the UV-dry and untreated titanium surfaces. These results suggest that the storage of titanium surfaces in dH_2_O could prevent further pollution of the titanium surface as caused by organic impurities in the air, with wet storage methods possibly maintaining the peak intensity of the TiO_2_ as observed immediately after the UV treatment.

### Decreased negative charges immediately and 28 days after UV treatment, with and without dH_2_O

**Figure 3:**
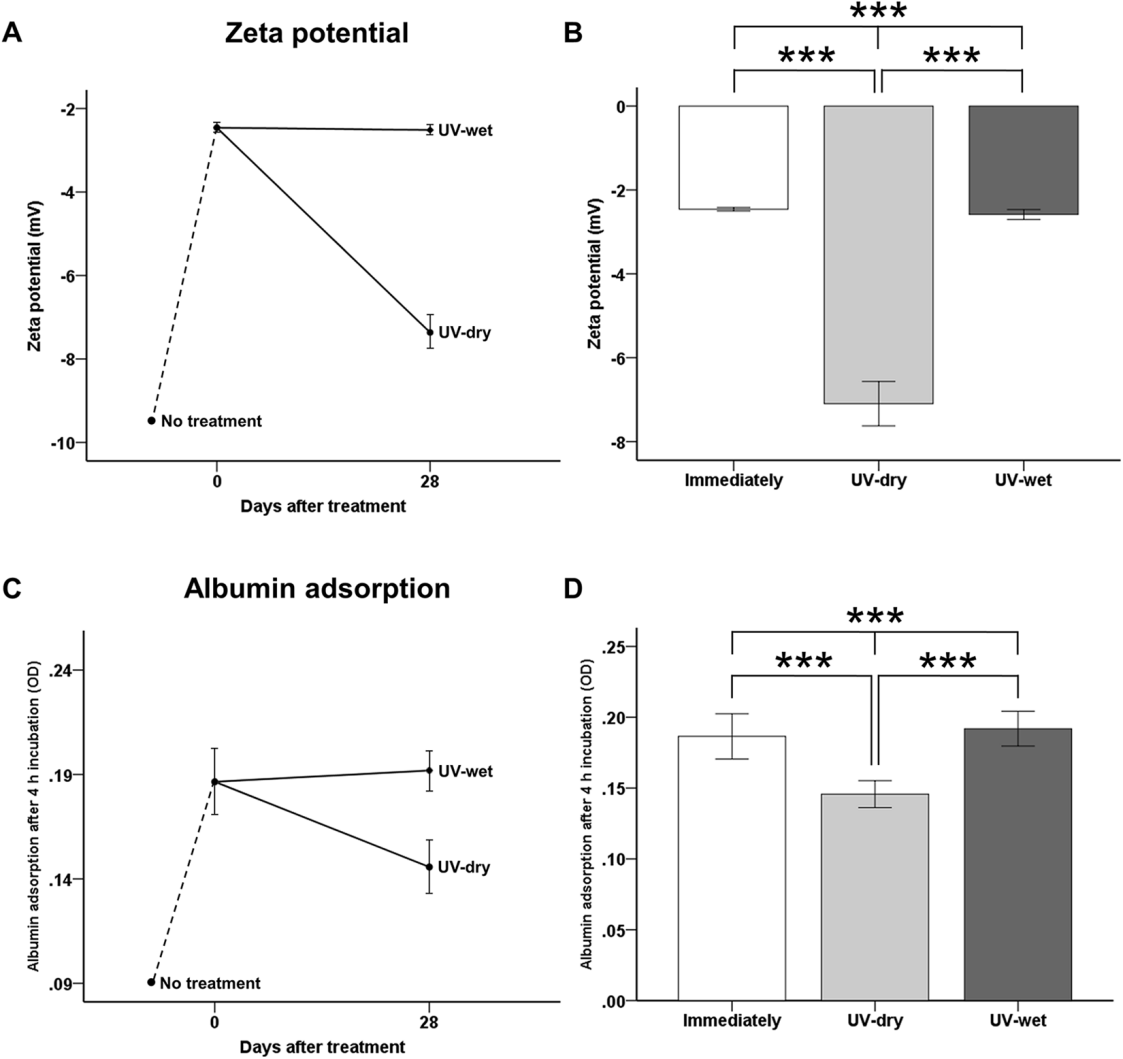
(**A**,**B**) Comparison of zeta potential of titanium disc surface after UV treatment, for each group at pH 7.4. (**C**,**D**) Comparison of optical density (OD) of albumin adsorption after UV treatment for each group. ***P < 0.001 for comparison between groups.

The zeta potentials increased immediately after UV treatment, from −9.59 ± 0.33 mV to −2.46 ± 0.43 mV at pH 7.4, and differed significantly between groups (*P* < 0.001; Fig. 3A,B). At 28 days after treatment, the zeta potential of the UV-dry titanium surface was markedly lower at −7.37 ± 0.36 mV, but that of the UV-wet titanium surface was almost the same −2.52 ± 0.01 mV. No significant differences were observed in the zeta potentials of the titanium surface immediately after UV treatment and UV-wet titanium surfaces (*P* > 0.05).

### Protein adhesion capacity immediately and 28 days after UV treatment, with and without dH_2_O

Immediately after UV treatment, the amounts of BSA adsorbed onto the titanium surface during the 4-h experimental period were significantly greater than those on the untreated titanium surface (*P* < 0.001; Fig. 3C,D). On the titanium surface immediately after UV treatment, the OD value of the BSA attached to the titanium discs increased from about 0.09 to 0.19. At 28 days after treatment, the OD value for the UV-dry titanium surface decreased to 0.15 ± 0.01, but that for the UV-wet titanium surface was almost the same 0.19 ± 0.01, indicating that the electrical polarity of the 28-day-old treated titanium disc surface in dH_2_O was sufficient to induce albumin adhesion to the titanium surface, while the protein adhesion capacity was comparable with that of the titanium surface immediately after UV treatment.

### Cellular adhesion capacity immediately and 28 days after UV treatment, with and without dH_2_O

**Figure 4:**
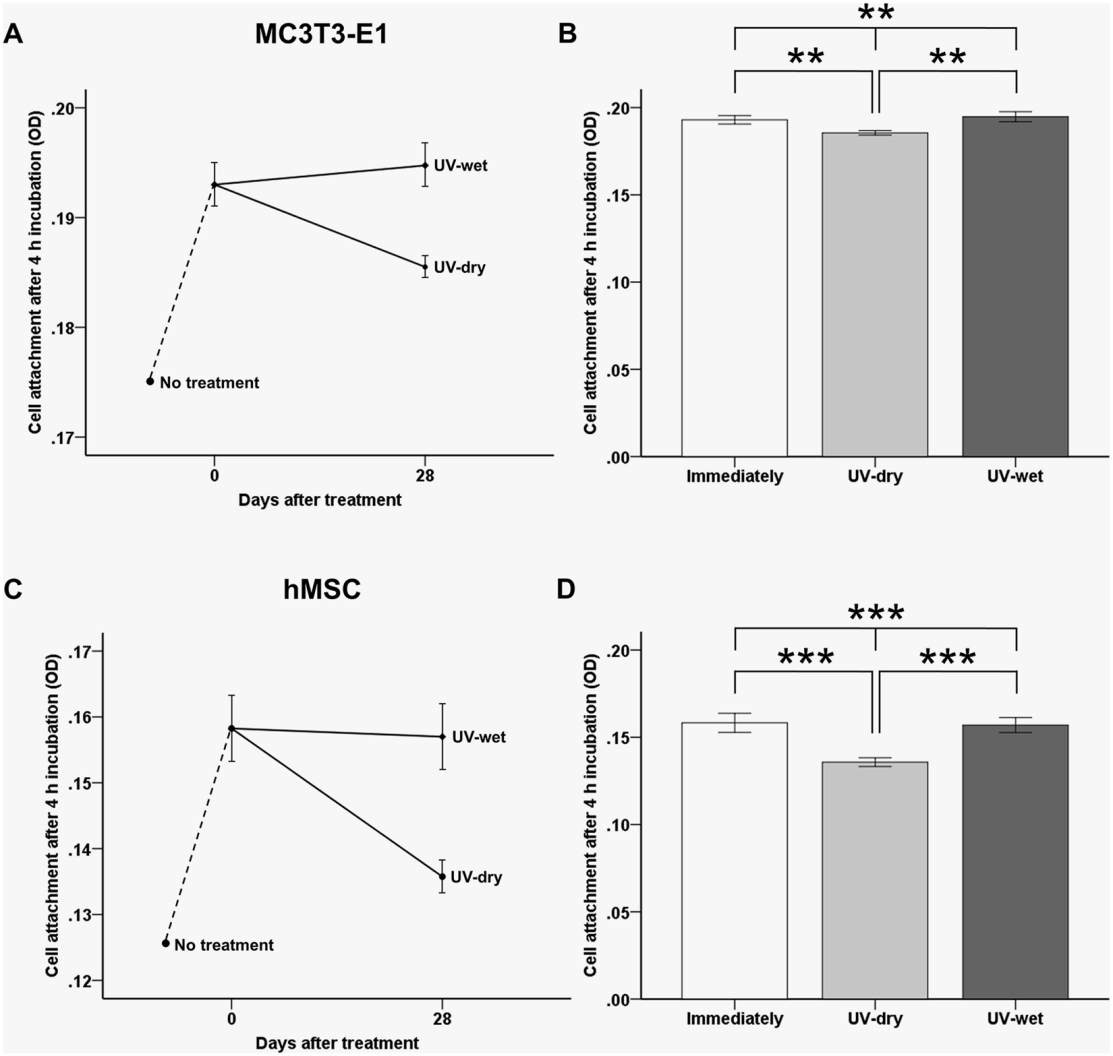
Comparison of optical density (OD) of MC3T3-E1 cell (**A**,**B**) and human mesenchymal stem cell (hMSC) attachments (**C**,**D**) on the titanium disc surface after UV treatment for groups after 4 h of incubation. **P < 0.01, ***P < 0.001 for comparisons between groups.

After 4 h of incubation, we found that the numbers of MC3T3-E1 and hMSC adherent cells had increased for the UV-treated samples, relative to the untreated titanium samples, regardless of whether they had been stored in dH_2_O (Fig. 4). We also found that the OD values for the MC3TC-E1 cell attachment on the titanium surface immediately after UV treatment had increased significantly from about 0.174 to 0.193 (*P* < 0.01) while the value was almost unchanged on the UV-wet titanium surface, being (0.195 ± 0.00), 28 days after treatment (Fig. 4A,B). Similarly, we found that the OD values for hMSC cell attachment on the titanium surface, immediately after UV treatment, had increased significantly from about 0.126 to about 0.158 (*P* < 0.001), while the value for the surface of the UV-wet titanium surface was almost unchanged (0.157 ± 0.00) 28 days after treatment (Fig. 4C,D), indicating that the 28-day-old treated titanium disc surface in dH_2_O could promote osteoblastic and mesenchymal cell adhesion to the titanium disc surface, while its cellular adhesion capacity was comparable to that of the titanium surface immediately after UV treatment, regardless of the cell type.

### Changes in cellular morphology immediately and 28 days after UV treatment, with and without dH_2_O

**Figure 5:**
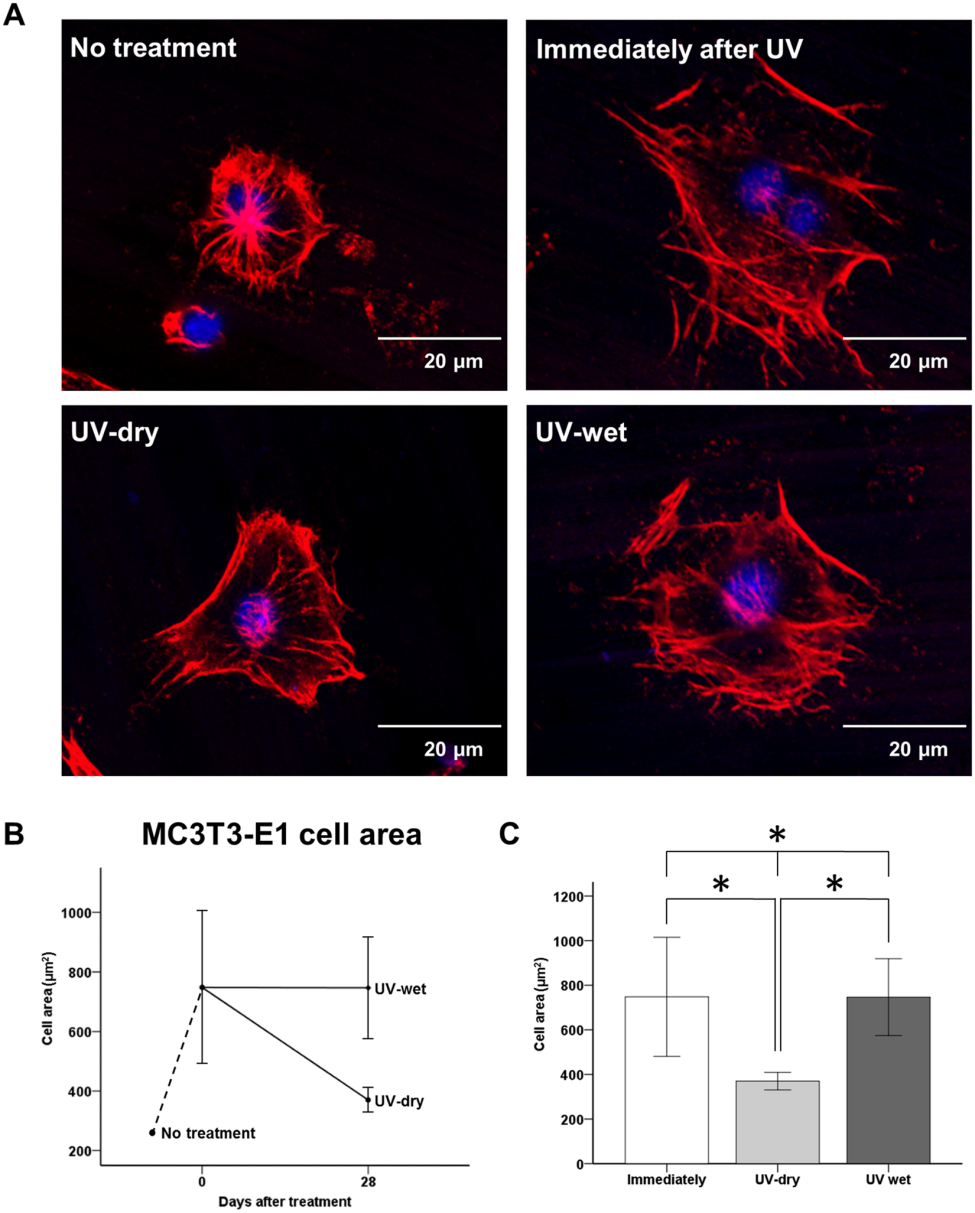
(**A**) Comparison of MC3TC-E1 cellular morphology on titanium disc surface after UV treatment for each group. (**B**,**C**) Comparison of cytoskeleton development such as cell area after UV treatment, for each group. *P < 0.05 for comparisons between groups.

After 4 h of incubation, we observed larger MC3T3-E1 cells with extended actin filaments and a spindle shape on the UV-treated titanium discs used immediately after treatment, relative to the untreated titanium surface, on which the cells were more circular (Fig. 5A). The mean cell area of the osteoblastic cells on the surface of the titanium disc immediately after UV treatment 748.00 ± 267.04 µm^2^ and on the UV-wet titanium surface 746.67 ± 172.29 µm^2^, 28 days after UV treatment, was significantly greater than on the UV-dry titanium surface 370.00 ± 39.51 µm^2^, 28 days after UV treatment (*P* < 0.05; Fig. 5B), indicating that the 28-day-old treated titanium disc surface in dH_2_O could enhance the osteoblastic cellular spread and cytoskeleton development, while its capacity was comparable to that of the titanium surface immediately after UV treatment.

### Changes in intracellular ROS levels immediately and 28 days after UV treatment, with and without dH_2_O

**Figure 6:**
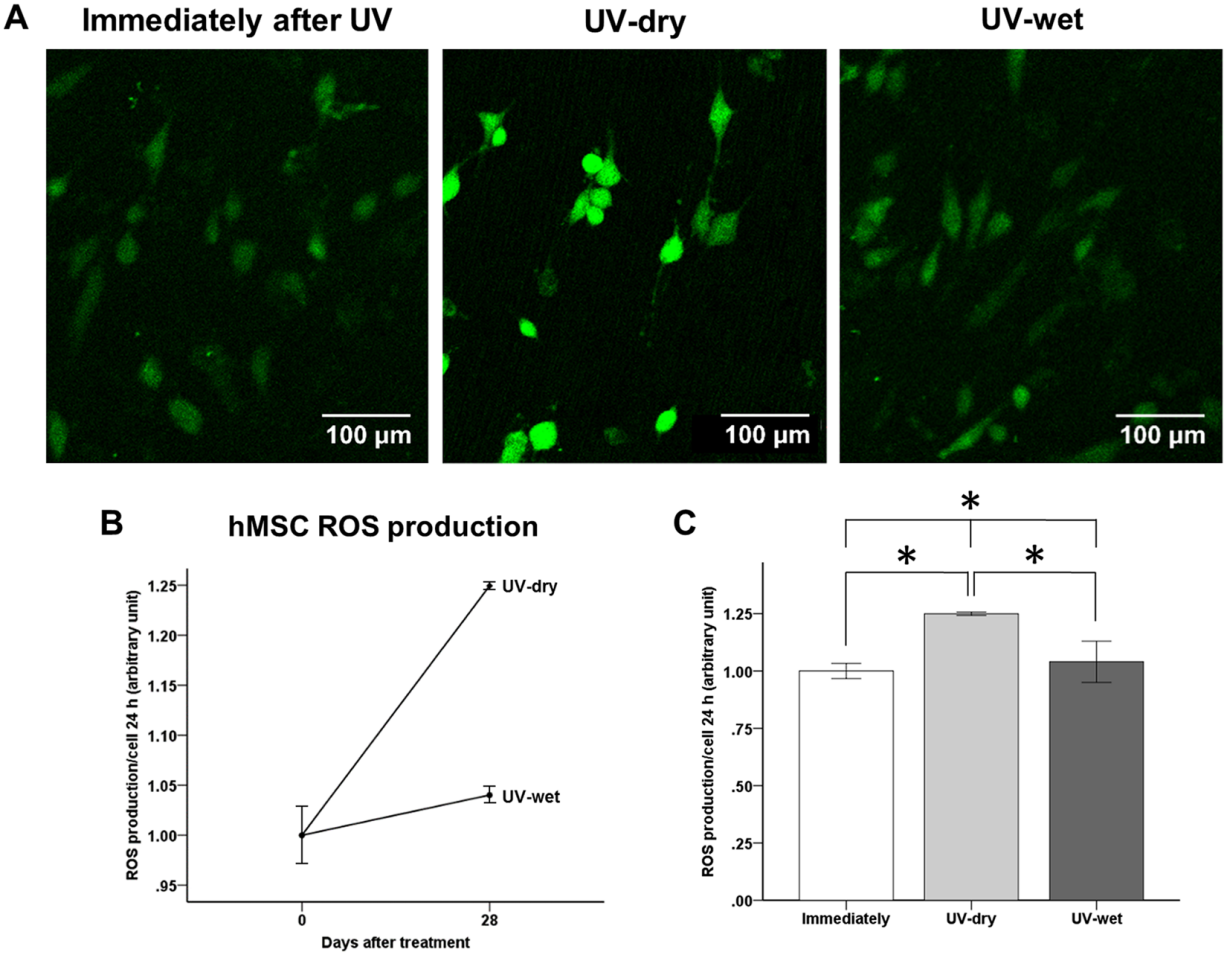
(**A**) Comparison of cellular intracellular reactive oxygen species (ROS) production of hMSC cells after UV treatment, for each group. (**B**,**C**) Comparison of intensity of intracellular ROS signal after UV treatment, for each group. *P < 0.05 for comparisons between groups.

After 24 h of incubation, the numbers of hMSC adherent cells on the titanium surface immediately after UV treatment and on the UV-wet titanium surface, 28 days after treatment, were significantly higher than those of the UV-dry titanium surface, 28 days after treatment. However, the ROS signal intensity was stronger in the cells on the UV-dry titanium surface (Fig. 6A). Fluorophotometric analysis showed that the ROS signal intensity of the UV-dry titanium surface, 28 days after treatment, was about 1.25 times greater than those of the titanium surface immediately after UV treatment and the UV-wet titanium surface, 28 days after treatment (*P* < 0.05; Fig. 6B,C), indicating that the 28-day-old treated titanium disc surface in dH_2_O could reduce the intracellular production of ROS, which inevitably occurs in mesenchymal cells in contact with the titanium surface. The oxidative stress of the cell on this surface was comparable with that of the titanium surface immediately after UV treatment.

## Discussion

In this study, we aimed to evaluate whether the biological activity of a titanium surface when stored in an aqueous solution after UV treatment is comparable to that of the surface immediately after UV treatment. This is because few studies have been conducted with the assumption that the biological activity of titanium after UV treatment can be maintained for a long period of time while in storage. This concept is applicable to the surfaces of all titanium implants used in the medical and dental fields, and therefore, has the potential to reduce the inconvenience to patients and clinicians.

Importantly, we found that there were no differences in the surface characteristics, surface zeta potential, protein adsorption, or cellular adhesion and oxidative stresses of MC3T3-E1 and hMSC cells on the titanium surfaces, both immediately after UV treatment and after being stored in dH_2_O for 28 days after UV treatment. These data provide important insights into the effects of the wet storage method on titanium implants for clinical applications.

In this study, the UV treatment did not alter the average surface roughness when analysed immediately after treatment. Nevertheless, this method increased the hydrophilicity of the titanium disc surface due to changes in the surface chemistry using two possible mechanisms: photocatalysis by UV-A (315–380 nm) and photolysis by UV-C (100–280 nm)^32^. Previous studies revealed that UV-A, which corresponds to a wavelength of approximately 365 nm, can induce TiO_2_ photocatalytic activity and excite an electron from the valence band to the conduction band through the application of 3.2 eV. These electron-hole pairs and electrons can lead to the generation of superoxides and ROS, such as the hydroxyl radical. An increased ROS number would induce a more hydrophilic state^33^. UV-C at a peak wavelength of 250 nm has also been used to improve the wettability of materials via photolysis, that is, the direct decomposition of hydrocarbon contamination^34^. Reducing the hydrocarbon level through the application of UV-C light is strongly associated with increased protein adsorption and cell attachment^35^. Although this study has not been able to clearly identify which wavelengths have the greater impact on the reduction of the carbon percentage of the titanium surface, it is assumed that that UV-A makes the greater contribution because the UV–vis absorption spectra for titanium surfaces mainly exhibit the absorption peaks at around 360 nm (339–374 nm), implying that the titanium may respond to a wavelength range of UV-A for the initiation of photocatalysis, for degrading hydrocarbons^13, 35, 36^.

To date, most previous studies investigating the effects of UV on hydrophilicity have not considered the shelf life or the consequent rehydrophobisation as biological aging of the titanium surface after UV treatment, which occurs rapidly under ambient storage conditions^9, 37^. In this study, the hydrophilicity of the UV-dry titanium surface under sterile humidified conditions without dH_2_O fell to almost half that of the untreated titanium surface, 28 days after UV treatment. As the peak corresponding to the carbon and the atomic percentage of carbon increased, the protein adsorption and cell adhesion capacity decreased over time because this hydrophobic, hydrocarbon-contaminated surface can change the surface zeta potential to an electronegative charge in the same was as serum albumin molecules and extracellular matrix proteins, while also causing the entrapment of air bubbles and blocking the RGD terminal, thus interfering with the interaction between the proteins and cells^7, 9, 38^.

Therefore, in this study, using commercially available UV-pretreated titanium implants, we focused on the UV treatment and water-based storage method as solutions for preventing rehydrophobisation because of contamination with hydrocarbon, while maintaining the surface hydrophilicity of titanium implants during long-term shelf storage. Wennerberg *et al*.^19^ reported that, after sand-blasting and acid etching, the hydrophilicity of the surface of titanium could be preserved by storage in a NaCl solution, while also preventing excessive chemical contamination from the atmosphere and prompting the reorganization of the outermost titanium oxide layer into a well-defined nanostructure. However, this has also been reported for the surfaces of titanium that has simply been stored in isotonic saline for an extended period without the exclusion of the influence of heterogeneous ions^39^. In this study, the UV-wet titanium surface, stored in dH_2_O for 28 days after UV treatment, was found to exhibit hydrophilicity and biological activity such as protein adhesion, osteoblastic and mesenchymal cell adhesion, and osteoblastic cellular cytoskeleton development that is comparable to those observed immediately after UV treatment. Additionally, the ROS signal intensity of the attached hMSC cells of the UV-wet titanium, 28 days after UV treatment, was found to be comparable to that of titanium immediately after UV treatment, but lower than that of UV-dry titanium, 28 days after treatment.

Oxidative stress induced by ROS overproduction hinders bone healing at the interface between the bone and implant because it adversely affects the cell growth on the implant surface but large numbers of ROS would inevitably be generated when the cells are in contact with a biomaterial surface such as titanium, or as a result of complex reactions during the surgical procedure^40–43^. To decrease the intracellular ROS production, although the administration of pharmaceutical antioxidants can be considered, conventional methods of drug administration to reduce oxidative stress-related defences face many challenges, such as the targeting of the bone repair cells and the cytotoxicity of antioxidant. Recently, Ueno *et al*.^17^ reported that intracellular ROS production can be limited by the UV treatment of titanium, while the reduced intracellular ROS level can contribute to improving the cytoskeleton development of osteoblast cells. Similarly, in this study, MC3TC-E1 cells on the UV-dry titanium, 28 days after UV treatment, exhibited a significantly reduced cytoskeleton development and degree of cell spread than other groups due to the biological aging of the titanium with contamination by hydrocarbons and, as expected, hMSC cells on the UV-dry titanium, 28 days after UV treatment, exhibited a significantly higher intracellular ROS signal intensity than the other groups. In contrast, the UV-wet titanium, stored in dH_2_O after UV treatment, maintained a favourable environment for the hMSC cells while producing almost the same titanium ROS signal immediately after treatment, indicating that this storage method may contribute to the promotion of bone-implant osseointegration due to the maintenance of the cellular oxidative stress, compared to immediately after the UV treatment.

Several limitations of this study should be considered when interpreting these data. In this study, because the titanium samples were flat discs, 12 mm in diameter, the results of this study may not be applicable to actual clinical settings, because the size of the titanium sample was much smaller than complex prosthetic orthopaedic implants, particularly hip or knee joint prostheses. Additionally, *in vitro* findings do not always translate to *in vivo* experience. We used a single-protein solution (BSA) to evaluate the adhesion of the protein to the titanium surface and therefore we did not account for competition between various proteins or human plasma. However, to evaluate any changes in the biological activity of titanium according to the presence or absence of wet storage immediately after UV treatment and storage, a study performed *in vitro* is a prerequisite. Based on the results of this study, a randomised controlled trial of exposure *in vivo* with compromised patients will be necessary to confirm whether these results can be applied to actual clinical situations in the medical and dental fields to ensure the long-term survival of the titanium implants.

Despite the limitations of this study, we found that there were no differences in surface characteristics, surface zeta potential, protein adsorption, or cellular adhesion and oxidative stresses of the MC3T3-E1 and hMSC cells, between the titanium surfaces immediately after UV treatment and those stored in dH_2_O for 28 days after UV treatment. The UV-instigated photocatalytic activity removed the surface hydrocarbons and altered the surface charge from negatively charged and hydrophobic (bioinert) to relatively positively charged and hydrophilic (bioactive); the surface characteristics produced by the UV treatment were relatively well maintained by the wet storage method for 28 days. Consequently, titanium stored in dH_2_O for 28 days after UV treatment enhanced blood protein adsorption, osteoblastic and mesenchymal cell attachment, and cytoskeleton development. Additionally, titanium stored in dH_2_O for 28 days after UV treatment created a favourable environment for mesenchymal cells by having a low oxidative stress due to the low ROS production, the level being like that of titanium immediately after UV treatment.

Therefore, UV treatment combined with a wet storage method could be one means of overcoming the biological aging of titanium, and this storage method can be applied to the practical application of UV-pretreated titanium implants. Future *in vivo* studies are necessary to confirm whether these results can be applied to actual clinical situations in the medical and dental fields.
